# Aerosol Azacytidine Inhibits Orthotopic Lung Cancers in Mice through Its DNA Demethylation and Gene Reactivation Effects

**DOI:** 10.1371/journal.pone.0109874

**Published:** 2014-10-27

**Authors:** Xuan Qiu, Yuanxin Liang, Rani S. Sellers, Roman Perez-Soler, Yiyu Zou

**Affiliations:** 1 Departments of Medicine, Division of Medical Oncology, Albert Einstein College of Medicine, Bronx, NY, United States of America; 2 Department of Pathology, Albert Einstein College of Medicine, Bronx, NY, United States of America; H.Lee Moffitt Cancer Center & Research Institute, United States of America

## Abstract

We devised an aerosol based demethylation therapy to achieve therapeutic efficacy in premalignant or in situ lesions of lung cancer, without systemic toxicity. Optimum regimens of aerosolized azacytidine (Aza) were designed and used in orthotopic human non-small cell lung cancer xenograft models. The therapeutic efficacy and toxicity of aerosol Aza were compared with intravenously administered Aza. We observed that 80% of the droplets of the aerosol Aza measured ∼0.1–5 microns, which resulted in deposition in the lower bronchial airways. An animal model that phenocopies field carcinogeneisis in humans was developed by intratracheal inoculation of the human lung cancer cells in mice, thus resulting in their distribution throughout the entire airway space. Aerosolized Aza significantly prolonged the survival of mice bearing endo-bronchial lung tumors. The aerosol treatment did not cause any detectable lung toxicity or systemic toxicity. A pre-pharmacokinetic study in mice demonstrated that lung deposition of aerosolized Aza was significantly higher than the intravenous route. Lung tumors were resected after aerosol treatment and the methylation levels of 24 promoters of tumor-suppresser genes related to lung cancer were analyzed. Aerosol Aza significantly reduced the methylation level in 9 of these promoters and reexpressed several genes tested. In conclusion, aerosol Aza at non-cytotoxic doses appears to be effective and results in DNA demethylation and tumor suppressor gene re-expression. The therapeutic index of aerosol Aza is >100-fold higher than that of intravenous Aza. These results provide a preclinical rationale for a phase I clinical trial of aerosol Aza to be initiated at our Institution.

## Introduction

Lung cancer claims 1.4 million lives every year (http://www.who.int/mediacentre/factsheets/fs297/en/index.html) [1], [2]. Lung cancer occurs as a result of cumulative damage in the bronchial epithelium caused by inhaled carcinogens. Because the cumulative damage affects the entire airway, damaged airway epithelium is prone to develop additional primary tumors during an individual’s lifespan [3]. All non-small cell lung cancers (NSCLC) originate from the bronchial epithelium covering large or small airways, giving rise to central or peripheral tumors. For example, squamous cell lung carcinomas most often arise centrally in large bronchi, lung adenocarcinomas typically develop peripherally in the smaller airways, and large cell lung carcinomas may arise in either location. However, all tumors originate from transformed airway epithelial cells. Therefore, selective therapeutic intervention for tumors confined to the airways should effectively inhibit or delay their formation without causing systemic toxicity [4]–[7]. Unfortunately, no therapies specifically targeting the bronchial epithelium are currently available.

Increasing knowledge in the field of epigenetics and carcinogenesis has led to the conclusion that aberrant epigenetic changes play an important role during lung carcinogenesis; moreover, these changes are maintained through the entire process of disease progression [8], [9]. For instance, silencing of tumor suppressor genes (TSGs) by aberrant hypermethylation has been found to play an important role in cancer initiation and development in multiple cancer types [10]–[19]. TSG promoter hypermethylation has also been shown to correlate with poor prognosis and resistance to chemotherapy [20]–[26]. In particular, all genetic lesions in lung cancers, including p53 and k-ras mutations, could be the consequence of aberrant epigenetic changes [10], [27], [28]. Epigenetic changes are reversible carcinogenic events [10], [29]. The cancer-specificity of these epigenetic changes makes them unique targets for specific epigenetic therapies [30]. Therefore, we hypothesize that by targeting the airway epithelium with a demethylating agent by aerosol administration, we may affect favorably the natural history of lung cancer.

Previously we observed that hypermethylation in the promoter region of the Rassf1 gene in human NSCLC cell line H226 can be reversed by the demethylating agent azacytidine (Aza). We also demonstrated, using a clinically relevant animal model developed by our lab, that intratracheal injection (a similar form of local administration as aerosol) of Aza at sub-toxic doses can increase the survival of mice orthotopically implanted H358 and H460 lung cancer [31], [32]. This clinically relevant animal model was proof of concept that airway targeted epigenetic therapy may have an advantage in the prevention and treatment of early NSCLC. Here we report on the efficacy of aerosolized Aza in the treatment of orthotopic human NSCLC xenograft models in mice as well as the efficacy of therapy on the demethylation of specific promoters of TSGs in tumor tissue. To elucidate the epigenetic therapeutic mechanisms, we resected tumor tissues from the xenografted animals treated with aerosol Aza, analyzed the methylation status of the promoters of 24 lung cancer-related TSGs, and determined the protein expression levels of those genes that showed significant promoter demethylation after aerosol Aza treatment.

## Materials and Methods

### Cell lines

Human NSCLC cell lines H226, H358, and H460 were purchased from American Type Culture Collection (ATCC) and cultured in RPMI-1640 medium supplemented with 10% fetal bovine serum (Invitrogen) and maintained in a 37°C incubator with 5% CO_2_ and 95% air.

### Animals

Male and female ICR and Athymic nude mice, 6–8 weeks old (Harlan) were housed in the animal facility at Albert Einstein College of Medicine. All animal studies were carried out in strict accordance with the recommendations in the Guide for the Care and Use of Laboratory Animals of the National Institutes of Health. The protocol (Protocol Number: 20130312) was approved by the Institutional Animal Care and Use Committee (IACUC) of Albert Einstein College of Medicine. In the survival study, the animals were observed daily. Humane endpoints were used during this study: moribund were humanely euthanized. We used two criteria to identify the moribund animals based on our animal use protocol: 1) mouse has difficulty breathing, eating, or drinking; 2) a mouse loses ≥15% body weight in 4 days. Mice were euthanized by CO2 in an inhalation chamber followed by exsanguination. Anesthesia was used to minimize discomfort during intratracheal injections.

### Intratracheal injection

For intratracheal (IT) injection, a bolus of cells in suspension or drug was injected into the trachea. The procedure used for IT injection has been previously described by us [33]. Briefly, mice are anesthetized by with an Isoflurane vaporizer at 2.5% isoflurane delivered by 2.0 PSI of oxygen and immobilized using a restraint device. The mouth is opened with a forceps and a small tubular light device is inserted into the mouth to locate the trachea, if needed. A 22 G feeding needle attached to a 1 ml syringe containing the cell suspension or drug solution is inserted into the trachea. Approximately 3 µl solution/g body weight is then injected intratracheally. The mouse is released from the restraining device at the completion of the procedure and observed until full recovery from anesthesia.

### Orthotopic endobronchial lung cancer xenograft models

Athymic nude mice were anesthetized as described previously and human NSCLC cancer cells in suspension were carefully inoculated into the trachea (approximately 2–4×10^6^ cells/mouse) using the method described above. In these models, multiple tumors grow within the pulmonary airways. The lifespan of the animals correlates with the tumor burden, which is related to the size of the inoculum.

### Aerosol formulation

Azacytidine (Sigma) was dissolved into sterile normal saline at 10 mg/ml right before dosing. The aerodynamic size of aerosol Aza was determined with extrusion-precipitation method using a 7-Stage Cascade Impactor linked to PARI’s nebulizer system per manufacturer’s instructions [34]. Aerosol administration. The aerosol was generated using a PARI’s personal compressor and LC Star nebulizer system. The aerosol generation rate was about 0.25 ml/min. A nose-only exposure system (CH Technologies Inc. Westwood, NJ) linked to the PARI’s aerosol system in a closed chemical hood was used for aerosol administration to mice. The aerosol administration time (min) (T) was strictly controlled. The aerosol dose deposited in the lungs (mg/m^2^) (D) was calculated by multiplying the time of exposure (min) by the aerosol inhalation rate (ml/min) (R) and the drug concentration (mg/ml) (C) as described previously by us [34], i.e. D = TRCI/W, where I is body surface/weight index (mg/m^2^ : mg/kg) (3 for mice) and W is animal body weight (kg). In a previous study, we measured aerosol inhalation rate in mice, defined as the volume of the aerosol liquid deposited in mouse lungs in 1 min was about 10^−4 ^ml/min for a 25 g mouse [34]. The desired deposited dose is achieved by controlling the aerosol time as T = DW/RCI. For example, the aerosol administration time for a deposited dose of 2.5 mg/m^2^ in a 25 gm mouse using a 10 mg/ml Aza concentration solution is T = 2.5×0.025/10^−4^×10×3 = 20.83 min.

### Toxicity studies

ICR mice (6/group) were treated with aerosol Aza at 2.5 or 75 mg/m^2^ daily×7 days. One group of mice was treated with IT Aza at the maximum tolerated dose of 270 mg/m^2^ as positive control for lung toxicity. The lungs were resected 7 days after the last aerosol administration. The lung toxicity was determined by pathological evaluation (3 lungs/group) using a previously described method [34]. Additionally, the viability of the airway epithelial cells was also determined (3 lungs/group). Briefly the lung tissue was digested with liberase (Roche) and filtered to obtain a single cell suspension, which was labeled with anti-mouse Ep-CAM antibody and fluorescence-conjugated goat anti-rat secondary antibody (Biolegend, San Diego, CA), and sorted by flow cytometry. The percentage of viable cells was determined by the exclusion of 4′, 6-diamidino-2-phenylindole staining. The systemic toxicity was determined by measuring blood cell counts as an indicator of myelosuppression (the major toxicity of IV Aza) [35]. Blood samples were taken at the time of euthanasia by cardiac puncture 7 days after last treatment. The total number of white blood cells (WBC) was determined after removal of red blood cells as previously described [32].

### Pre-pharmacokinetic study

Normal ICR mice were given aerosolized Aza at 2.5 mg/m^2^ using the method described above. At each designed time point (5 min, 20 min, 2 h, 6 h and 24 h), three mice were euthanized and their lungs were resected after right ventricle perfusion with saline. Azacytidine in the lungs was extracted and quantitatively measured by a previously reported method using LC-MS system [36]. The quantitative detection was performed by Millis Scientific Inc. The sensitivity was 1 ng Aza/ml of sample. The Aza concentration in tissue vs. time data were analyzed and simulated with the best fitting (the highest R^2^ value) under the Microsoft Excel program. The equation of the simulated curves C = f(t) were used to calculated AUC from 0 to 24 hours as AUC = ∫_0–24_ f(t) dt. Peaking time (T) and peak concentration were obtained directly from observation of the curves.

### Antitumor efficacy

Ten days after the intratracheal inoculation of tumor cells, the nude mice were randomly divided into 3 groups (6 mice/group): 1) aerosolized Aza at 2.5 mg/m^2^ daily for 7 days, 2) aerosolized normal saline (vehicle) daily for 7 days, 3) or IV Aza at 75 mg/m^2^ daily for 7 days. In this survival study the animals were observed daily. Humane endpoints for euthanasia (previously described) were used during this study and moribund mice were humanely euthanized and mice were necropsied. The Increased lifespan (%ILS) [37] was used to assess the efficacy of each tested agent.

### Histology of lung cancer xenografts

Mice from the anti-tumor efficacy study were necropsied after death and lung and tumor samples for histology were collected and placed into 10% neural buffered formalin. Samples were transferred to 70% ethanol and submitted to the Histology and Comparative Pathology Shared Resource at Einstein for routine processing to paraffin. Tissues were sectioned to 5 µm, stained with hematoxylin and eosin (H&E), and evaluated by a board certified veterinary pathologist.

### Analysis of methylation status in lung tumors

Mice bearing orthotopic lung tumors were divided into 3 groups and treated with aerosol Aza, aerosol vehicle, or IV Aza. Two weeks after the final treatment, the mice were euthanized and visible lung tumors (1∼3 mm) were resected, frozen on dry ice, and broken down into smaller (<0.5 mm) pieces by an eppendorf-tube homogenizer. Each tumor sample was divided into two aliquots: one for methylation assay and another for western blot. The methylation status of the promoter regions of 24 tumor suppressor genes involved in lung carcinogenesis was measured using a methylation qPCR array method by Qiagen (SA Bioscieces) as previously described [38].

### Analysis of protein expression in lung tumors

The second aliquot of the lung tumor samples was used to determine protein expression of the TSGs. The TSGs showing significant promoter demethylation after aerosol Aza treatment as analyzed by the methylation qPCR array were selected for evaluation by western blot. The frozen tissues were homogenized in cold RIPA buffer with protease inhibitor cocktail (Sigma) using a hand homogenizer, followed by sonication. Protein samples were prepared for Western blot following a routine protocol from Life technologies (Grand Island, NY). Primary antibodies against human H-cadherin, OPCML, Rassf1a (Abcam), SFRP1 (Epitomics, Burlingame, CA), and beta-actin (Santa Cruz Biotechnology) were used to identify the targeted proteins. LI-COR C-DiGit Blot Scanner was used to scan the western blots; the bands were analyzed by Image Studio software (LI-COR). The density ratio of targeted protein vs. actin loading control of each sample was used to present the protein expression levels semi-quantitatively.

### Statistical analysis

Differences among different groups were analyzed by two-side Log-rank (Mantel-Cox) test. A difference was considered statistically significant when p<0.05.

## Results

### Orthotopic NSCLC models

**Figure 1:**
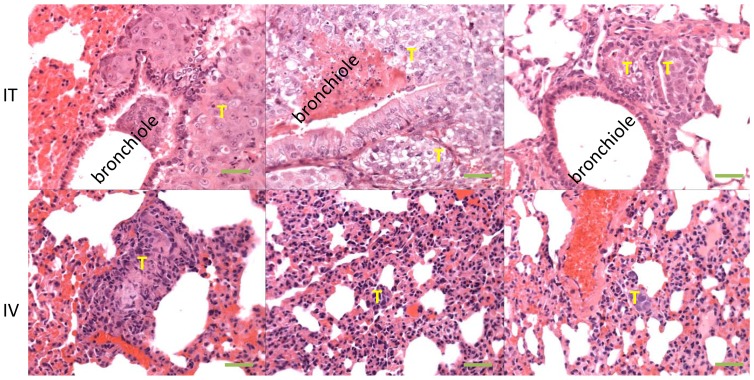
Orthotopic NSCLC model. The model was created by intratracheally injecting NSCLC H460 cells into nude mice. Top: H & E stained lung sections from IT inoculated mice on day 35. The tumors (T) arise within the airways and grow vicinally into the lung parenchyma. Bottom: H & E stained lung sections from the IV inoculated mice on day 35. Tumors arise within small vessels in the lung parenchyma. The objective magnification was 40X. The scale bars were 50 µm.

NSCLC is a disease of the airway epithelium as a result of chronic exposure of airborne carcinogens. In order to mimic this particular tumor growth in the airway in a xenograft model, we intra-tracheally inoculated human NSCLC cells directly into the airways, and then compared this mode of transplantation to IV injection of tumor cells into nude mice. Histological examination of the lung revealed that the tumor distribution and growth pattern was more similar to human lung cancer in the IT implantation model: the majority of the cancer cells seed on the surface of the airway epithelium at approximately one week after implantation. By 3 weeks, small mucosal tumor nodules were localized within the airway or the lung tissue adjacent to the airway, indicating primary tumor growth within the airway and invasion to the lung parenchyma (Figure 1 top row). The tumor growth and size are cell line- and inoculum size- dependent. In our experience, H460 (large cell carcinoma) tumors developed faster than H358 (bronchioalveolar carcinoma) and much faster than H226 (squamous cell carcinoma) when inoculated IT in mice. In the absence of any intervention, mice succumb to the lung tumors between days 40 and 102. There were no identifiable distant metastases. In contrast, almost all visible/detectable IV implanted cancer cells seeded in the capillary system of the lung and relatively far away from the airway (Figure 1 bottom row), more closely resembling metastatic tumor cells migrating from other organs to the lung. The tumors also developed faster than after IT implantation, and the mice usually died on days 35 to 68 (data not shown). Our results suggest that IT implantation is a more appropriate orthotopic xenograft model for mimicking human NSCLC than the IV model or other ectopic models.

### Aerosol formulation

**Figure 2:**
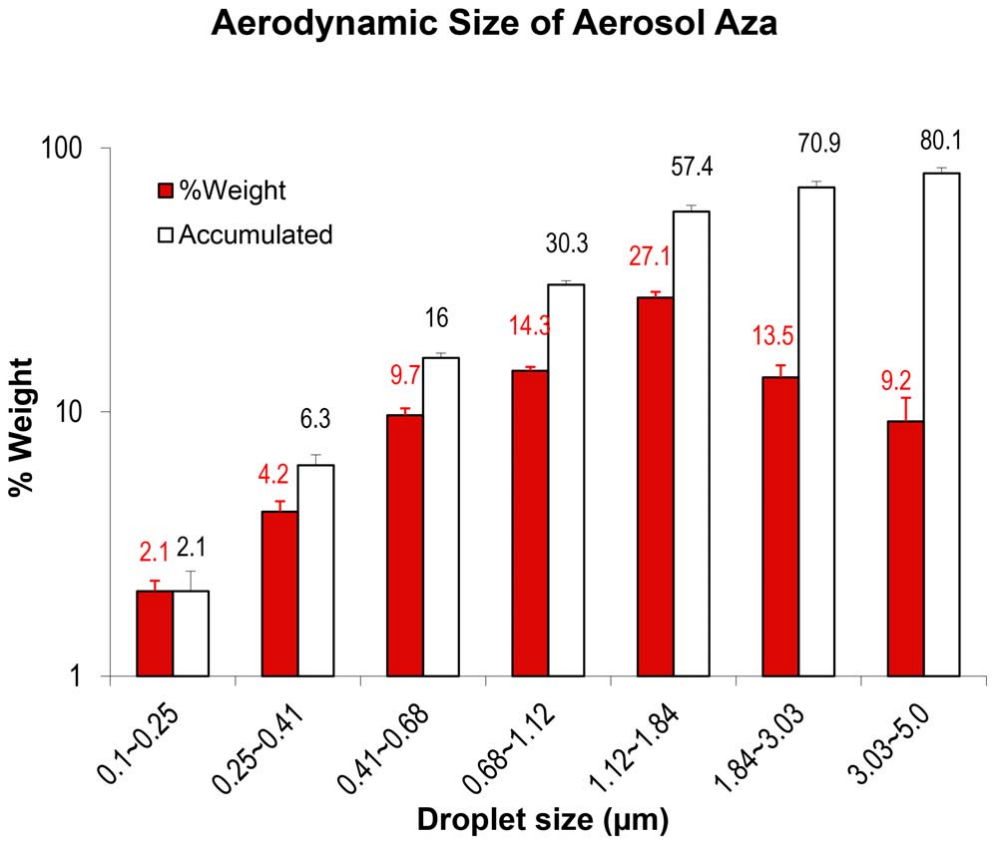
Aerodynamic size of Aerosol Aza. T determined with extrusion-precipitation method using a 7-Stage Cascade Impactor linked to PARI’s personal compressor and LC star nebulizer system. Aza solution (4 ml) was aerosolized at an airflow rate of 5 L/min. The condensed aerosol samples were collected at 3 different intervals, from 1 to 1.5 min, from 3 to 3.5 min, and from 5 to 5.5 min. Aerodynamic size and fraction of aerosol with a particular size range were measured and calculated as per manufacturer’s protocol. The data was mean ± standard deviation of the aerodynamic size based on weight (solid bars) and cumulative weight (empty bars) from 3 independent experiments.

We have developed an aerosol formulation for the prototypical demethylation agent, Azacytidine (Aza). The formulation is composed of Aza and injection grade sterile normal saline or water. The Aza power can be quickly dissolved (<30 sec) in the aqueous solution just prior to aerosol administration. It has been suggested that aerosol droplets <5 µm, particularly <3 µm, in diameter deposit most frequently in the lower airways and, therefore, are appropriate for pharmaceutical inhalation of aerosol preparations by humans [39] [40]. We measured the aerodynamic size of the Aza aerosol formulation, under our aerosol generation system with the extrusion-precipitation method [34], and found that about 80% of the droplets (weight%) were between 0.25–5 µm in diameter, and about 71% between 0.25∼3 µm (Figure 2). Our results suggest that about 71∼80% of the droplets should deposit in the distal airway in humans [39] [40].

### Toxicity and dose selection

**Figure 3:**
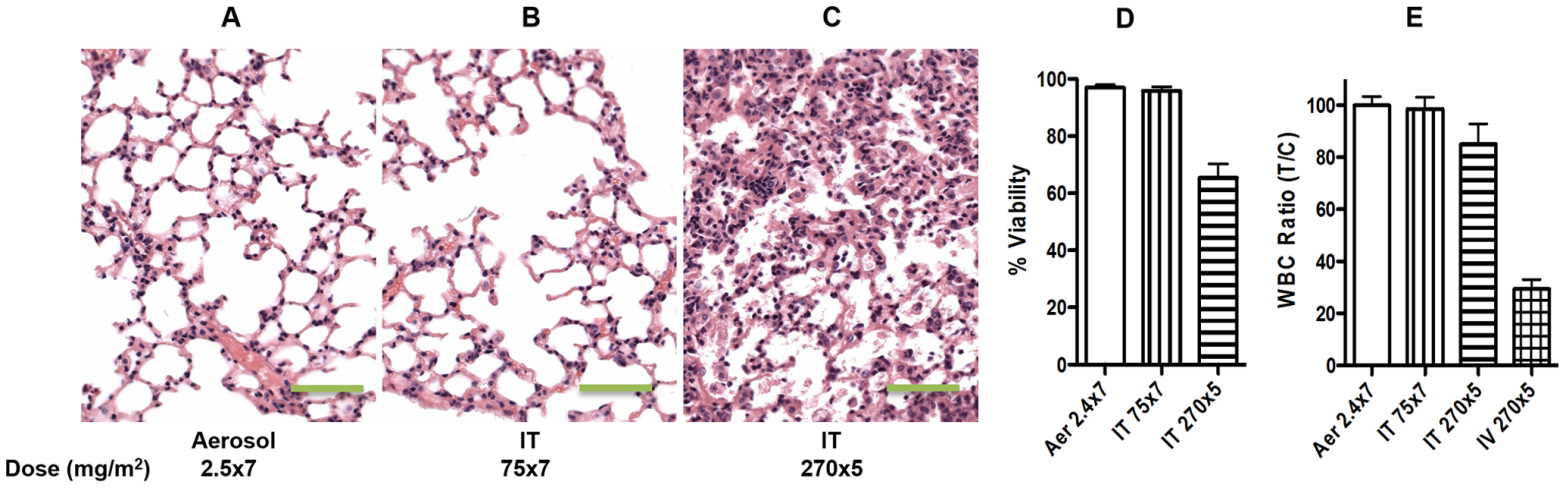
Toxicity of aerosol Aza. Lung sections of mice treated with aerosol Aza at 2.5 mg/m^2^×7 (A), IT Aza at 75 mg/m^2^×7 (B), and IT Aza at 270 mg/m^2^×5 (C), respectively show no toxicity at 2.5 and 75 2.5 mg/m^2^ but pneumonitis at 270 mg/m^2^. The objective magnification was 40X. The scale bars were 50 µm. White blood cell count ratio (after treatment vs. before treatment, n = 6) (D) and percentage of viable cells of the sorted airway epithelial cells (n = 6) (E), respectively.

From our previous study we have learned that the effective demethylation concentration of Aza for cultured cells could be 3 logs lower than its IC_50_. The antitumor effective dose (7.5 mg/m^2^×3) by intratracheal injection of Aza in the mouse model did not cause lung toxicity or detectable systemic acute toxicity, and lung toxicity only occurred at the highest dose of 270 mg/m^2^, which was the maximum tolerated dose by IV injection [32]. Based on these results, we selected a dose for aerosol Aza that we predicted would have demethylation function and therapeutic efficacy without significant toxicity. The results indicate that the selected aerosol dose of 2.5 mg/m^2^ (about 21 min aerosol exposure to a 25 g mouse) daily for 7 days or a higher intratracheal bolus injection dose of 75 mg/m^2^ for 5 days did not cause any detectable lung toxicity (evaluated histologically) or systemic toxicity (measured by myelosuppression) on day 7 after the final administration (Figure 3A, B). Pulmonary toxicity (pneumonitis) (Figure 3C) and myelosuppression (Figure 3D) only occurred when the mice received the highest dose of intravenous or intratracheal injection of Aza at 270 mg/m^2^ (the MTD of intravenous Aza). Similarly, the aerosol Aza at the selected therapeutic dose did not cause any death of airway epithelial cells determined by flow cytometry, as compared to the positive control in mice treated with the highest IT doses of Aza at 270 mg/m^2^ (Figure 3E).

### Therapeutic efficacy

**Figure 4:**
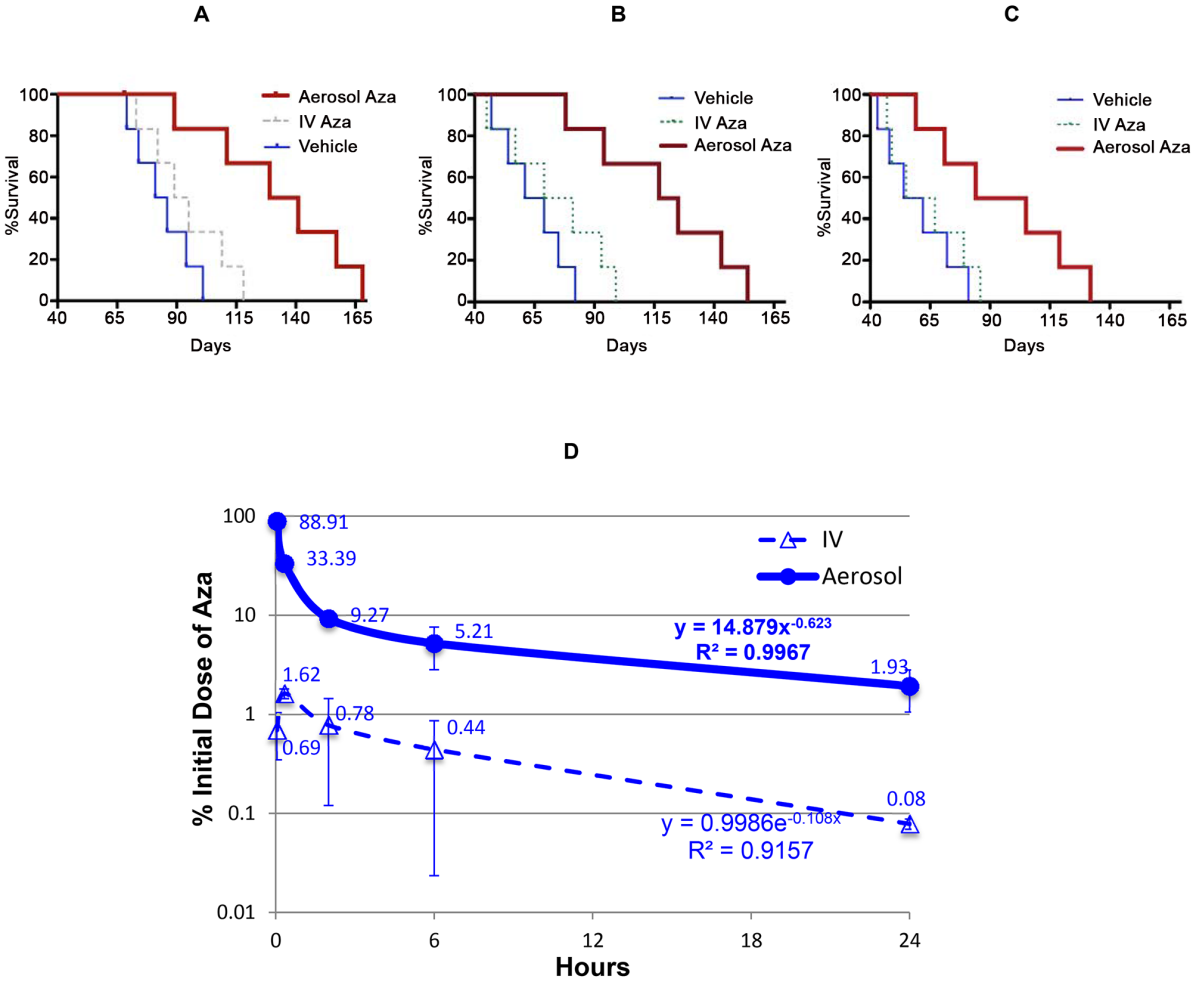
Aerosol administration of Aza significantly prolongs the survival of mice with orthotopic human NSCLC xenografts (A∼C). Mice intratracheally inoculated with the cell lines H226 (A), H358 (B), or H460 (C) were treated with aerosol Aza (red thick line) at 2.5 mg/m^2^ daily for 7 days or aerosol vehicle (blue thin line) with the same volume. Treatment with IV Aza (dash line) at the optimal dose of 75 mg/m^2^ daily for 5 days was used as control. The statistical comparison of the survival curves was performed with Log-rank (Mantel-Cox) test. The p values for H266, H358, and H460 models were 0.0135, 0.0150, and 0.0719, respectively. Pre-pharmacokinetics of the aerosol Aza (D). ICR mice were treated with aerosol Aza at 2.5 mg/m^2^. The data at each time point was the average ± standard deviation of the percent of given/initial dose from lungs of 3 mice. The equation for each curve was simulated curve with the best fitting (the highest R^2^ value) under the Microsoft Excel program.

Systemic (IV) administration of Aza has led to only limited efficacy in NSCLC patients [41] [42], which has been recapitulated in our experimental mouse models of NSCLC treated with IV Aza, (Figure 4A–C). There is actually little experimental evidence supporting the use of a demethylation agent to effectively inhibit lung cancer in animal models [43] [32]. One of the possible reasons is that this very unstable drug was not efficiently delivered to the lung tumors. We were the first group to report a proof of concept study in which the administration of Aza directly into the airways effectively prolonged the survival of mice with orthotopic lung cancers [32]. In the current study, we are reporting the results using aerosol Aza for the treatment in human orthotopic NSCLC xenografts in mice. One of our goals was to demonstrate that aerosol delivery of Aza to the airway epithelium could effectively inhibit the growth of clinically relevant NSCLC models. In addition, these studies were further designed to investigate whether aerosol delivery of the demethylating agent could inhibit the growth of tumors within the airways at low demethylation doses rather than cytotoxic doses (Figure 4A–C).

Our results indicate that a low dose aerosol Aza significantly prolonged the lifespan of the mice bearing endobronchial tumors. The %ILS and median survival were prolonged by 5.1- and 1.5-, (p = 0.0135), 6.4- and 1.9- (p = 0.0150), and 8.9- and 1.6- (p = 0.0719) fold in the groups treated with aerosol Aza using a dose more than 20-fold lower than the dose used in the systemic treatment control group, in the H226, H358, and H460 model, respectively. These results clearly demonstrate that aerosol administration of Aza at demethylating, non-cytotoxic doses is superior to systemic administration of Aza in terms of tumor growth inhibitory efficacy.

### Pre-pharmacokinetics

To determine the deposition of Aza in the lungs we conducted pre-pharmacokinetics studies in vivo. Intravenously injected Aza with the same dose was used a comparison. At the first time point (3 min, the earliest possible time point), we could recover ∼89% of the initial aerosol dose from the lung. However, when the same dose was given by systemically injection, the peak concentration in the lungs was only 1.62% of the initial dose. The area under the concentration vs. time curve (AUC_0∼24 h_) of aerosol Aza was 15-fold larger than that of IV Aza (Table 1). Aza given via both routes showed a similar decline pattern: a rapid decline within the first 2 h after the administration followed up by a slower decline. Aerosolized Aza showed a slower rate of decline in the slower phase while IV Aza showed a delayed peaking concentration in the lung (Figure 4D). These data demonstrate that the aerosolized Aza has higher tissue concentration in the lungs with predictable lower systemic exposure than the IV Aza.

**Table 1:** Pre-pharmacokinetic parameters.

	T (min)	C_max_ (%ID)	AUC_0∼24 h_ (%ID^.^h)
**IV**	20	1.62	9.45
**Aerosol**	3	88.9	146
**Aero vs. IV**	0.15	55	15

T is peaking time in minute. C_max_ is peak concentration of azacitidine presented as percent of given/initial dose (%ID). AUC is the area under the concentration vs. time curve from 0 to 24 h.

### Aerosol Aza demethylation

**Figure 5:**
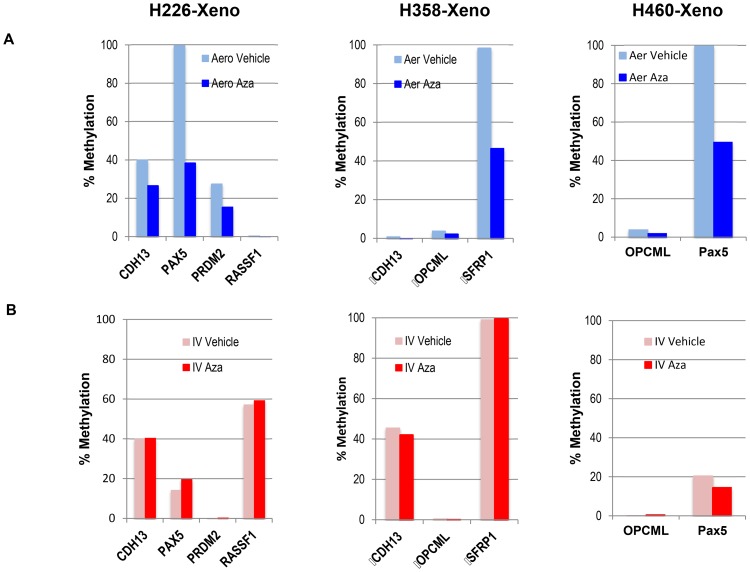
Methylation changes in the lung tumors. Tumor-bearing mice from 3 orthotopic lung cancer models (H226-xeno, H358-xeno, and H460-xeno) were treated with aerosol Aza (A) or IV Aza (B) at 2.5 mg/m^2^ daily×7. The lungs were resected 14 days after the final treatment and tumor nodules larger than 1.5 mm were isolated for methylation detection by qPCR array technology at Qiagen (SA Biosciences). The data is %methylation of the total CG cytosine of the particular promoter region.

To analyze whether Aza has a demethylating effect in the clinically relevant NSCLC models, we harvested the lung tumors from the xenograft models treated with aerosol Aza or IV Aza and determined the methylation status of the promoter regions of 24 tumor suppressor genes, whose promoter hypermethylation has been reported as relevant in the lung cancer literature. Normal bronchial epithelial cells from animals without Aza treatment were used as methylation baseline control. The 24 TSGs selected are APBA1, APC, CADM1, CDH1, CDH13, CDKN1C, CDKN2A, CDKN2B, CXCL12, CYP1B1, DLC1, FHIT, MLH1, MTHFR, PAX5, PRDM2, RARB, RASSF1, RASSF2, SEMA3B, SFRP1, SLIT2, TCF21, and TGFB1. We found that 11 of the 24 promoters were hypermethylated in the tumor samples from 3 different xenograft models and that aerosol Aza could demethylate 5 of them (Figure 5A). In contract, IV Aza at the same dose did not show any demethylating effect (Figure 5B). The data shown in Figure 5 depict methylation level detected by a q-PCR methylation array and are presented as %methylation of the total CG cytosine of the particular promoter region.

### TSG re-expression

**Figure 6:**
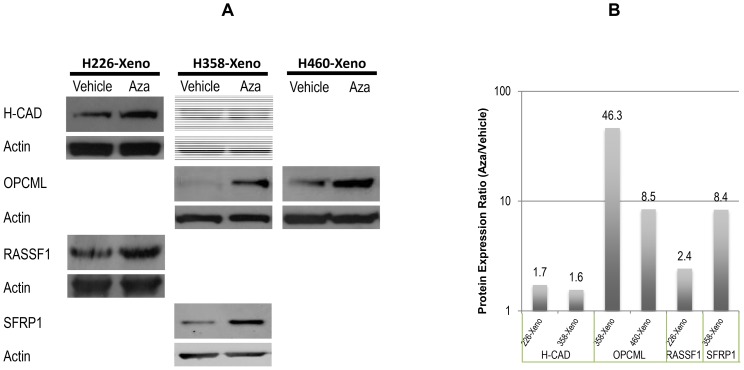
Protein expression of the TSGs in the lung tumors (A). Western blotting assay was used to determine the protein expression of the TSGs with significant promoter demethylation after aerosol Aza treatment identified by the methylation q-PCR array. The tumor samples were the same as used in Figure 5; Histogram of the Western blots (B). The western blot photo films were scanned and the density ratio of targeted protein vs. actin loading control of each sample was presented as the protein expression level.

In order to analyze whether the demethylation at the promoter region of TSGs can reactivate the TSGs, we resected the lung tumors and measured the protein expression of the TSGs whose promoters were significantly demethylated after the aerosol Aza treatment. Western blotting assay was used to detect the gene expression in the tumor samples from the same mice used in the demethylation study. We selected 5 TSGs whose promoters were significantly demethylated after the aerosol Aza treatment. We found that H-Cad and Rassf1 in the H266 tumors, H-cad, OPCML, and SFRP1 in H358 tumors, and OPCML in H460 tumors were reactivated at the protein level (Figure 6A and B) after the aerosol Aza treatment. Expression of these TSGs has been shown to inhibit or delay the cancer development in several types of cancers including lung cancer. For instance, we have reported before that loss of H-cadherin in human NSCLC is associated with tumorigenicity [44]. The promoter region of Rassf1a is also found heavily methylated in lung cancer [45]. OPCML, a broad tumor suppressor originally found in ovarian cancers, is found methylated in many lung cancer cell lines [46]. SFRP1 in the Wnt signaling pathway is transcriptionally silenced by promoter hypermethylation in NSCLC [47]. The results of this in vivo study imply that aerosol demethylating treatment may be effective in inhibiting endo-bronchial lung tumors and bronchial premalignant lesions.

## Discussion

Lung cancer is viewed as a “genetic disease” [48], [49]. Upon the demonstration that TSG deactivation and oncogene activation play a critical role in carcinogenesis, the therapeutic efforts have progressively shifted from optimizing the non-specific radiation and chemotherapy forms of therapy that mainly kill fast-dividing cells to develop agents that target the specific genetic changes. Recent evidence suggests a direct relationship between aberrant epigenetic changes and cancer development; it indicates that cancer is also in part an epigenetic disease. Unlike genetic changes, epigenetic changes occur earlier in cancer progression and are reversible. Therefore, a therapeutic strategy aiming at reversing aberrant epigenetic changes should be a more effective strategy than those focusing on the use of non-specific cytotoxic agents or on targeting irreversible genetic defects.

According to the field cancerization model [3] [50], all bronchial epithelial cells accumulate epigenetic and genetic lesions until one single cell acquires a malignant phenotype and gives rise to an invasive tumor, while the remaining of the field continues accumulating epigenetic and genetic damage and is at risk of developing other primary lung cancers (second primaries) [51] [52]. During this process, the aberrant epigenetic changes, such as hypermethylation of the promoters of TSGs, occur prior to and may cause the subsequent genetic changes, and they persist in tumor cells through the entire process of carcinogenesis.

On this basis, a strategy based on targeting the reversible aberrant epigenetic changes of the epithelial cells in the whole airway epithelium should be very effective in delaying or preventing primary and subsequent lung tumors. To test his hypothesis, we inoculated human lung cancer cell lines to mice by intratracheal injection. Tumor implantation within the airspace leads to tumor growth from the site where the cells are arrested, either large and/or small airways, and are thus more clinically relevant than other xenograft models implanted subcutaneously, injected intravascularly, or injected directly into the lung parenchyma. Therefore, it mimics more closely the whole airway field cancerization model and is also ideally suited to test the efficacy of aerosol treatments. The aerosolized Aza tested in our studies was effective in prolonging the lifespan of animals bearing tumors within the airways, thus strongly suggesting that this method has the potential to inhibit or prevent lung cancer tumors that arise in the bronchial epithelium of individuals exposed chronically to airborne carcinogens.

Our results also demonstrate that direct delivery of demethylating agents to the bronchial epithelium is a rational molecular strategy to reverse the aberrant hypermethylations associated with the lung carcinogenesis process. Using our orthotopic lung cancer mouse model we have showed that aerosol administration of Aza at non cytotoxic doses results in reactivation of several key TSGs through demethylating their hypomethylated promoters in lung tumor cells that are accessible by administration by aerosol. This constitutes experimental proof of principle that a single demethylation agent delivered by aerosol can inhibit orthotopic lung cancer at non-cytotoxic dose, and the results of pre-pharmacokinetic study demonstrated that the aerosolized drug deposits more efficiently in the lungs, and this is potentially one of the reasons for the higher efficacy in reactivating TSGs and antitumor activity with lower systemic toxicity. In addition, these effects were observed at a dose of Aza that is much lower dose than that used in the clinic and therefore without detectable local lung or systemic toxicity.

The lung carcinogenesis process involves very complicated epigenetic and genetic alterations affecting the entire genome. Aberrant methylation is one of the important epigenetic alterations that can lead to carcinogenesis. Based on current evidence, the majority of cancers have aberrant hypermethylation in the promoters of TSGs, although the whole genome may appear to be hypomethylated [53]. Our study was limited to 24 promoters, and the results could only identify methylation changes in a small number of the potential targets relevant to lung cancer. Ongoing efforts in our laboratory are focused on whole genome methylation screening using additional animal models to look at spontaneously occurring lung tumors as well as inhaled carcinogen-induced lung cancer. These studies are expected to yield much more valuable information on the biology of lung carcinogenesis, which should guide the development of improved therapeutic strategies.

Reed et al. have recently reported a similar study using aerosol Aza for the treatment of an orthotopic lung cancer model in nude rats [54]. They concluded that their aerosol and systemic deliveries are equally effective in terms of antitumor efficacy as well as gene demethylation [54]. Although the methodologies, tumor models, and endpoints used in Reed study and our study are significantly different, the fact that both studies lead to some similar conclusions using aerosol Aza represents strong proof of concept of the potential of aerosol demethylation therapy as a novel strategy for the management of lung cancer and bronchial premalignancy.

In our study, we found that the effective dose was much lower than the optimal IV dose, indicating that targeted aerosol delivery of the minimal effective dose rather than the maximum tolerated dose of an epigenetic agent is a potentially effective therapeutic strategy. In addition, our aerosol formulation showed a very good aerodynamic size range that makes it suitable for use in humans. We have recently completed preclinical toxicity studies of aerosol Vidaza in mice and FDA approved us to initiate a Phase I clinical study with aerosol Vidaza in patients with advanced lung cancer with disease mostly confined to the lung parenchyma that are not candidates for therapies of a higher priority. In this study, in addition to determining the toxicity profile of aerosol Vidaza, we will investigate by sequential bronchoscopy whether aerosol Vidaza can lead to TSG reexpression in the bronchial epithelium and or/tumor tissue. We expect that the results of this study will determine whether inhaled demethylation therapy should be further developed both as an early intervention or even prevention strategy. If that is the case, significant effort will have to be devoted to explore the use of other candidate agents as Aza is potentially carcinogenic [55] and therefore not an ideal candidate as a preventive agent.
